# Social dysfunction in Ecuadorian, Peruvian, and Venezuelan media workers

**DOI:** 10.1371/journal.pmen.0000709

**Published:** 2026-09-02

**Authors:** Byron Fernando Bustamante-Granda, Claudia Torres-Montesinos, Jhon Remigio Espinosa-Iñiguez

**Affiliations:** 1 Grupo de Investigación Bienestar, Salud y Sociedad, Escuela de Psicología y Educación, Universidad de Las Américas, Quito, Ecuador; 2 Departamento de Psicología, Universidad Técnica Particular de Loja, Ecuador; PLOS: Public Library of Science, UNITED KINGDOM OF GREAT BRITAIN AND NORTHERN IRELAND

## Abstract

The personal, occupational and psychosocial risk and protection factors associated with social dysfunction in media workers are analyzed. A total of 314 workers were surveyed. Data were collected on personal and occupational variables, and scales were administered to assess coping styles, perceived loneliness, emotional impact of the health emergency, and social dysfunction, which was measured using Scale C of the General Health Questionnaire (GHQ-28). It was found that 19.7% were at risk of social dysfunction. This risk was particularly evident among workers without a formal partner and those performing fieldwork in peripheral cities. Bivariate analyses indicated that workers at risk were more likely to be women, younger, less experienced, with limited training during the crisis, and reported poorer health, lower perceived ability to cover risky events, greater concern about infecting their families with COVID-19 or dying, and a stronger desire to quit their jobs. Psychosocially, they experienced greater emotional impact, higher levels of loneliness, greater use of self-blame and behavioral disengagement, and less use of active coping. The final logistic regression model correctly classified 89.8% of cases. Factors associated with increased odds of social dysfunction included desire to change jobs, perception that health would not improve, concern about infecting their families with COVID-19, hyperconnectivity, and use of planning. Factors associated with lower odds included working in the capital city, seeking emotional support, and employing humor. The importance of preventing social dysfunction to improve the mental health and general well-being of media workers in crisis contexts is discussed. In summary, the social dysfunction in media workers increases with personal or family affectation, the desire to change employment, hyperconnectivity, planning, and self-blame, and decreases among those who work in the capital, seek emotional support, and use humor.

## Introduction

Social dysfunction is a person’s difficulty functioning in their daily lives, including problems with performing everyday tasks, maintaining the usual pace of work, enjoying normal activities, making decisions, performing their roles properly and fulfilling their responsibilities effectively and satisfactorily [1]. Operationally, it has been measured through subscale C of the General Health Questionnaire-28 (GHQ-28) [2] where a high score is related to problems of adaptation and motivation [1,3].

Social dysfunction has received scarce attention as a mental health problem in media professionals [4]. Most research has focused on problems such as substance use [5], burnout [6], depression [7] or post-traumatic stress disorder [4]. Mental health studies largely concentrate on field-based journalists, while office-based media workers, such as editors, designers, and media owners, remain underrepresented.

However, there are studies on field-based workers (e.g., journalists) who cover dangerous contexts and report high scores for social dysfunction (i.e., exceed the 5/6 cut-off point) [8,9]. Contrary to expectations, other studies have reported that scores for social dysfunction were below the cut-off point [10,11]. Only one study identifies a 2% risk of social dysfunction in a mixed sample of field‑ and office‑based media workers, using the 5/6 cut‑off point [12].

Complementarily, social dysfunction in media workers could be influenced by organizational changes, such as intensified demands and work stress, especially in media contexts where the conditions for practicing journalism have deteriorated [13]. The Latin American context is one of those that shows the greatest challenges to practice journalism safely [14] especially in peripheral cities, which are more exposed to censorship and self-censorship [15].

Certain subgroups of media workers may experience greater social dysfunction, particularly those covering front‑line assignments in wars, emergencies, protests, or disasters, where exposure to traumatic events is common [10,16–18] and where trauma prevention or support interventions are often inadequate [19]. These psychosocial risk factors are especially relevant in Latin American media, where a lack of protection and persistent job insecurity are common [15,20–22].

Also, other personal and work factors may be related to social dysfunction in media workers; among them: age is inversely related to the presence of mental health problems [12,23], the level of training for risk coverage [24], the conditions under which they telework [24,25], harassment [24,26], the desire to change jobs [12,24] and excessive workload [27].

Personal and family-related consequences of occupational exposure may also be associated with greater social dysfunction among media workers. In this regard, it has been reported that media workers who faced crises present somatizations and a worse perception of their health [28]. In the media, there was also an increase in cases of mourning for colleagues who died or suffered sequelae related to risk coverage [23]. Media work can also pose direct or perceived threats to family safety, including harassment or infecting family members due to exposure during the pandemic, as reported by health professionals [17,24,27] or, in the most severe circumstances, the murder of journalists covering corruption, drug trafficking, or politically sensitive topics [29]. These situations potentially generate greater family distress associated with the dangers of media work, and reinforcing the need for media workers to engage in adaptive coping strategies to manage these stressful life events.

A prevalence higher than 2% of social dysfunction among media workers would therefore be expected [12], because empirical studies suggest that, in contrast to anxiety and insomnia, levels of social dysfunction remain relatively stable over time when comparing the period of trauma exposure with journalist’s current condition [9] and this can alter the workers’ life projects and the organizational structure of media organizations [12,24]. In the media sector of developing countries, there are also difficulties related to receiving training and to accessing timely and evidence-based mental health prevention and intervention, which could make the psychological impact on workers chronic [30].

To improve the preparation of the media in the prevention of mental health problems among media workers, it is also necessary to understand their strategies for coping with stress, that is, the various psychological resources intended to manage stressful situations that can significantly influence their mental health [31]. In media workers, it has been reported that they use passive and maladaptive stress coping strategies (e.g., substance use) that could increase their risk of social dysfunction [5,28,31]. It is therefore necessary to encourage the use of stress coping strategies aimed at solving problems and strengthening support from family members, colleagues and supervisors to promote healthy adaptation [7,24,27,31,32]. It is also specifically recommended to stimulate social support to reduce the mental health impact resulting from isolation and the difficulties arising from adapting to teleworking [25,33,34]. Thus, identifying protective coping strategies is key to informing mental health care initiatives in the media sector.

However, there is currently no research identifying the risk or protective factors linked to social dysfunction among media workers in Latin America, across both field-based and office-based positions. The present study seeks to fill this gap by analyzing personal, occupational, and psychosocial determinants that may guide the development of evidence‑based training and preventive interventions.

## Materials and Methods

### Ethics Statement

The protocol was approved by the expedited committee on human research ethics (CEISH) of the Ministry of Public Health of Ecuador (Code: 009–2020) and the CEISH of the Pontifical Catholic University of Ecuador (Code: PV 05–2022). The collection of information from the participants ensured confidentiality and anonymity through the signing of informed consent. The data were collected between September 10, 2020, and January 06, 2021, in Ecuador, and between March 15 and September 30, 2022, in Ecuador, Peru and Venezuela.

### Objective

To identify personal, occupational, and psychosocial risk and protective factors associated with social dysfunction in media workers in Ecuador, Peru, and Venezuela.

### Participants

A non-probability convenience sampling strategy was employed. A total of 1105 invitations were sent by email and social media to complete a computerized survey. A total of 509 partial and complete responses were recorded (acceptance rate = 46.06%). After data cleaning and application of the eligibility criteria, 314 media workers from Ecuador (49%), Peru (19.4%) and Venezuela (31.6%), with an average age of 37.69 years +10.87, an average work experience of 12.88 years +10.18. Of these, 51.3% were women, 36.9% resided in the capitals, 64.7% did not live with a formal partner, 9.6% belonged to ethnic minorities, and 69.7% declared themselves middle class or higher. Of the participants, 65.6% were field-based workers (e.g., journalists, reporters, photographers) and 34.4% were office-based staff (e.g., editors, directors, producers, designers, columnists, web programmers, community managers, cartoonists, illustrators). Of these, 68.2% worked in digital media and 31.8% in traditional media (e.g., radio, television, or written press). Participants received no economic compensation for their participation.

### Measures

**General Health Questionnaire – Scale C (GHQ-28)**: The primary outcome was risk of social dysfunction (Risk = 1; No risk = 0) which was measured through the C scale of the GHQ-28 [2]. This instrument, adapted to Spanish, assesses social dysfunction through seven items, for which the participant responds using a Likert-type scale of four levels that were qualified as follows: 1 and 2 = 0, and 3 and 4 = 1. To obtain the total score of the scale, the points obtained across in the seven questions are added, and a participant is considered at risk when 6 or more points are obtained.

**Ad hoc surveys:** To collect the personal and work factors of the workers, a survey was applied that evaluated six dimensions: 1) Demographic: age, years of experience, sex, place of residence, marital status, ethnicity, socio-economic level, type of role. 2) Job training: risk coverage, characteristics of COVID-19, biosecurity, self-care in mental health. 3) Skills to cover crises: perception of aptitude to cover risk, difficulty in seeing a corpse. 4) Relationship with bosses and employment: desire to change jobs or leave journalism, perception of whether supervisors care about safety and well-being. 5) Perception of health impact: current health compared to the period prior to the health crisis and future health compared to health at the time of answering the protocol. 6) Stressors during the crisis: overwork, care work, housework, personal contagion, concern about infecting the family with COVID-19, losing the job, dying in the crisis, economic situation, and hyperconnectivity in personal and work life.

Three scales were applied to assess psychosocial factors:

1) The Perception of Social Support through the **University of California, Los Angeles Revised Short Loneliness Scale (UCLA-10)** [35]. It contains 10 items, which are answered on a four-level Likert scale (4: I never feel that way, 3: I rarely feel that way, 2: I feel that way sometimes, 1: I feel that way often). The 10 item scores are summed to yield a total score between 10 and 40; lower total scores reflect higher levels of perceived loneliness.
2) The 14 strategies for coping with stress were assessed through the **Brief-COPE test** [36]. It consists of 28 questions and evaluates 14 strategies for coping with stress: active coping, planning, emotional support, instrumental support, religion, positive reframing, acceptance, denial, humor, self-distraction, self-blame, behavioral disengagement, venting, and substance use. Each item is answered on a four-level Likert scale (0: I have not been doing this at all, 1: a little, 2: a moderate amount, 3: a lot), Each scale consists of two items, which are summed to obtain scores ranging from 0 to 6, with higher scores indicating greater use of the strategy.
3) The emotional impact of the health emergency was assessed through **the Brief scale of stress severity due to the perception of the COVID-19** [37]. This scale consists of three items, which are answered on a five-level Likert scale (0: unaltered, 1: slightly altered, 2: moderate alteration, 3: significant alteration, 4: severely altered). Similarly, the scores of the three items are added to obtain total scores ranging from 0 to 12, with higher scores indicating greater emotional impact of the COVID-19 emergency.

### Design and procedure

A cross-sectional study was conducted. The instrument battery was piloted, and the final versions for each country were approved at meetings of the research team. The instrument battery was digitized in the True Test data collection and storage software. The invitation letter, along with the access link, were sent by the research team via email, and invitations were also distributed by media executives and through the contact bases and social networks of the organizations participating in the study. In the software, informed consent was first presented, which could be revoked at any time. Completing the protocol required approximately 30 minutes. At the end, each participant received a report with their results and an invitation for a feedback interview, a self-care workshop, and, if they wished, psychological support sessions. During the application, no questions were mandatory, and no financial compensation was offered for participation.

### Data analysis

Only participants who completed 100% of the questions were included in the analysis. After filtering the data, descriptive analyses of measures of central tendency and dispersion were performed for continuous variables, and frequencies and percentages were calculated for categorical variables. In the bivariate analyses of continuous variables, the Mann-Whitney test was used, and for categorical variables the Chi-square test (χ^2^) was applied. The significance value was set at p < 0.05.

To determine the variables that predict social dysfunction, the variable was dichotomized: scores from 0 to 5 were classified as no risk of social dysfunction, and scores of 6 and 7 as risk of social dysfunction and a binary logistic regression was calculated, introducing the variables as follows: Layer 1 - demographic variables, job training, skills to cover the crisis, perception of health impact, stressors during the crisis, and relationship with bosses and employment. Layer 2 – coping strategies were added. Layer 3 – the perception of loneliness and the emotional impact of the health emergency were added. In each layer, the following were reported: Nagelkerke’s R^2^, Hosmer and Lemeshow goodness-of-fit test, log-likelihood, percentage of cases identified by the equation, and variables identified in the equation with the odds ratios (OR) and the 95% confidence intervals. The statistical analysis was carried out using the IBM Statistical Package for the Social Sciences (SPSS) software (IBM Inc., Chicago, IL, USA; version 25.0).

## Results

### Prevalence of social dysfunction and bivariate analyses

Overall, 19.7% of participants (n = 62) met the criterion for risk of social dysfunction (scores >6), and the mean was 2.77 + 2.28. The Mann-Whitney *U* test reported that workers at risk of social dysfunction are characterized by being younger (*U* = 5514.0; p < .001) and having less work experience (*U* = 5493.0; p < .001). The Chi-square test identified that workers at risk of social dysfunction are associated with the female sex (𝜒^2^ = 6.824; p = .009). Place of residence, marital status, ethnicity, socio-economic level and type of role were not significant (Table 1).

**Table 1:** Bivariate association: Social dysfunction and demographic factors.

		Risk of social dysfunction (n = 62)	No risk of social dysfunction (n = 252)	
		Mean (SD / Mean Rank)	Mean (SD / Mean Rank)	*U*
Age		32.90 (7.40 / 120.44)	38.87 (11.27 / 166.62)	5514.0*
Years of experience		8.52 (6.30 / 120.10)	13.96 (10.67 / 166.70)	5493.5*
		f (%)	f (%)	χ^2^
Sex	Male	21 (33.9)	132 (52.4)	6.824*
	Female	41 (66.1)	120 (47.6)	
Place of residence	Peripheral cities	41 (66.1)	157 (62.3)	0.313
	Capital	21 (33.9)	95 (37.7)	
Marital status	With partner	23 (37.1)	88 (34.9)	0.102
	Without partner	39 (62.9)	164 (65.1)	
Ethnicity	Mestizo	58 (93.5)	209 (82.9)	4.580
	White	2 (3.2)	15 (6.0)	
	Other minorities	2 (3.2)	28 (11.1)	
Socio-economic level	Low	4 (6.5)	11 (4.4)	1.91
	Medium-low	14 (22.6)	66 (26.2)	
	Medium	40 (64.5)	154 (61.1)	
	Medium-high	4 (6.5)	20 (7.9)	
	High	0 (0)	1 (0.8)	
Type of role	Office-based workers	15 (24.2)	93 (36.9)	3.563
	Field-based workers	47 (75.8)	159 (63.1)	

Note: * p < 0.05, SD = Standard deviation

The Chi-square test on labor factors found that workers who are at risk of social dysfunction are associated with not having received training on COVID-19 (𝜒^2^ = 5.635; p = .018) and biosecurity (𝜒^2^ = 5.420; p = .020), being perceived as less suitable for risk coverage (𝜒^2^ = 23.126; p < .001), and having more desire to change their jobs and leave journalism (𝜒^2^ = 21.254; p < .001) compared to risk-free workers (Table 2).

**Table 2:** Bivariate association: Social dysfunction and labor factors.

			Risk of social dysfunction (n = 62)	No risk of social dysfunction (n = 252)	
			f (%)	f (%)	χ^2^
Job training	Risk coverage	Yes	10 (16.1)	70 (27.8)	3.556
		No	52 (83.9)	182 (72.2)	
	Characteristics of COVID-19	Yes	8 (12.9)	69 (27.4)	5.635*
		No	54 (87.1)	183 (72.6)	
	Biosecurity	Yes	10 (16.1)	78 (31.0)	5.420*
		No	52 (83.9)	174 (69.0)	
	Self-care in mental health	Yes	5 (8.1)	33 (13.1)	1.184
		No	57 (91.9)	219 (86.9)	
Skills to cover crises	Ability to cover crises	Not apt at all	6 (9.7)	15 (6.0)	23.126*
		Unfit	27 (43.5)	45 (17.9)	
		Apt	23 (37.1)	125 (49.6)	
		Very apt	6 (9.7)	67 (26.6)	
	Difficulty in seeing a corpse	Nothing	11 (17.7)	69 (27.4)	3.343
		Little	27 (43.5)	99 (39.3)	
		Moderate	13 (21.0)	54 (21.4)	
		A lot	11 (17.7)	30 (11.9)	
Relationship with bosses and employment.	Desire to change jobs or leave journalism	Nothing	15 (24.2)	111 (44.0)	21.254*
		Little	16 (25.8)	87 (34.5)	
		Moderate	20 (32.3)	37 (14.7)	
		A lot	11 (17.7)	17 (22.5)	
	My supervisors care about safety and well-being	Nothing	18 (29.0)	48 (19.0)	3.788
		Little	17 (27.4)	64 (25.4)	
		Moderate	21 (33.9)	109 (43.3)	
		A lot	6 (9.7)	31 (12.3)	

Note: * p < 0.05.

The Chi-square test on the stressors associated with the health crisis found that workers who are at risk of social dysfunction are associated with perceiving that the crisis had a greater impact on their current health (𝜒^2^ = 10.056; p = .007) and future health (𝜒^2^ = 9.130; p = .010), being more concerned about infecting their family with COVID-19 (𝜒^2^ = 14.827; p < .001) or about dying during the health crisis (𝜒^2^ = 4.782; p = .029) compared to risk-free workers (Table 3).

**Table 3:** Bivariate association: Social dysfunction and stressors.

			Risk of social dysfunction (n = 62)	No risk of social dysfunction (n = 252)	
			f (%)	f (%)	χ^2^
Perception of health impact	Current vs pre-crisis	Better than March 2020.	4 (6.5)	50 (19.8)	10.056*
		Same as March 2020	25 (40.3)	115 (45.6)	
		Worse than March 2020	33 (53.2)	87 (34.5)	
	Current vs future	Better than now.	19 (30.6)	128 (50.8)	9.130*
		Same as now.	39 (56.5)	108 (42.9)	
		Worse than now	8 (12.9)	16 (6.3)	
Stressors during the crisis.	Overwork	Yes	27 (43.5)	112 (44.4)	0.016
		No	35 (56.5)	27 (43.5)	
	Care work	Yes	10 (16.1)	40 (15.9)	0.002
		No	52 (83.9)	212 (84.1)	
	Housework	Yes	10 (16.1)	36 (14.3)	0.135
		No	52 (83.9)	216 (85.7)	
	Personal contagion	Yes	18 (29.0)	64 (25.4)	0.341
		No	44 (71.0)	188 (74.6)	
	Concern about infecting the family with COVID-19	Yes	31 (50.0)	63 (25.0)	14.827*
		No	31 (50.0)	189 (75.0)	
	Losing the job	Yes	29 (46.8)	103 (40.9)	0.711
		No	33 (53.2)	149 (59.1)	
	Dying during the crisis	Yes	8 (12.9)	13 (5.2)	4.782*
		No	54 (87.1)	239 (94.8)	
	Economic situation	Yes	44 (71.0)	176 (69.8)	0.30
		No	18 (29.0)	76 (30.2)	
	Hyperconnectivity in personal and work life.	Yes	20 (32.3)	64 (25.4)	1.195
		No	42 (67.7)	188 (74.6)	

Note: * p < 0.05.

Mann-Whitney *U* tests indicated that workers at risk of social dysfunction were characterized by having a greater emotional impact associated with the health crisis (*U* = 5529.5; p < .001) and greater loneliness (*U* = 5262; p < .001). In addition, workers at risk of social dysfunction used less active coping strategies (*U* = 6531.0; p = .041), more self-blame (*U* = 5469.5; p < .001) and more behavioral disengagement (*U* = 5866.5; p = .001) compared to risk-free workers (Table 4).

**Table 4:** Bivariate association: Social dysfunction and psychosocial factors.

		Risk of Social Dysfunction (n = 62)	No risk of social dysfunction (n = 252)	
		Mean Rank	Mean Rank	*U*
Affectivity	Perception of loneliness	116.37	167.62	5262.0*
	Emotional impact of the health crisis	194.31	148.44	5529.5*
Coping strategies	Active coping	136.84	162.58	6531.5*
	Planning	174.91	153.22	6732.5
	Emotional support	155.05	158.10	7660.0
	Instrumental support	152.37	158.76	7494.0
	Religion	151.53	158.76	7442.0
	Positive reframing	159.82	156.93	7668.0
	Acceptance	157.10	157.60	7787.0
	Denial	162.95	156.16	7474.0
	Humor	151.20	159.05	7421.5
	Self-distraction	163.38	156.05	7474.5
	Self-Blame	195.28	148.20	5469.5*
	Behavioral disengagement	188.88	149.78	5866.5*
	Venting	163.96	155.91	7411.5
	Substance use	169.20	154.62	7086.5

Note: * *p* < 0.05.

### Binary logistic regression analysis

Predictors were entered hierarchically in theoretically defined blocks, in the following order:

Layer 1 – Includes demographic variables, job training, skills to cover crises, perception of health impact, stressors during the crisis, and relationship with bosses and employment. The model achieved an R^2^ = .436 (χ^2^(8) = 1.693, p = .989) and explained 86.6% of cases. The following risk factors were included in the model: desire to change jobs or leave journalism at a high level vs. nothing (OR = 4.075, 95% CI [1.410, 11.773]), and moderate versus nothing (OR = 5.034, 95% CI [1.301, 19.470]); perception of future health at the same level as now vs better than now (OR = 2.549, 95% CI [1.061, 6.121]); concern about infecting the family with COVID-19 (OR = 4.463, 95% CI [1.816, 10.972]) and hyperconnectivity in personal and work life (OR = 2.783, 95% CI [1.110, 6.986]). No protective factors were identified (Column 2, Table 5).

**Table 5:** Layered binary logistic regression.

Factor	OR / CI 95%	OR / CI 95%	OR / CI (95%)
Desire to change jobs or leave journalism (Nothing = 0)			
Moderate	4.075 [1.410, 11.773]	4.622 [1.197, 17.847]	4.458 [1.145, 17.352]
A lot	5.034 [1.301, 19.470]	–	–
Perception of future health (Better = 0)			
Same as now = 1	2.549 [1.061, 6.121]	3.740 [1.188, 11.774]	3.784 [1.150, 12.450]
Concern about infecting the family with COVID-19 (No = 0)			
Yes = 1	4.463 [1.816, 10.972]	6.540 [2.177, 19.649]	6.355 [2.084, 19.380]
Hyperconnectivity in personal and work life (No = 0)			
Yes = 1	2.783 [1.110, 6.986]	4.165 [1.341, 12.934]	4.198 [1.351, 13.050]
Place of residence (Peripheral cities = 0)			
Capital = 1	–	.335 [.118, .952]	.345 [.121, .989]
Coping strategy for stress: Emotional support	–	.533 [.315, .902]	.533 [.314, .903]
Coping strategy for stress: Humor	–	.648, [.452, .928]	.655 [.456, .941]).
Coping strategy for stress: Planning	–	2.259 [1.400, 3.644]	2.216 [1.371, 3.584]
Coping strategy for stress: Self-blame	–	1.419 [1.022, 1.970]	1.415 [1.004, 1.996]
Hosmer and Lemeshow goodness-of-fit test	χ2(8) = 1.693, p= .989	χ2(8) = 4.766, p= .782	χ2(8) = 6.511, p= .590
Log-likelihood	211.239	176.800	176.010
Nagelkerke’s R^2^	0.436	0.556	0.558

Note: CI = Confidence interval; OR= Odd ratio.

Layer 2- The 14 stress coping strategies were added. This model improved R^2^ to  .556 (χ^2^(8) = 4.766, p = .782) and accounted for 87.3% of cases. The protective factors were: 1) living in the capital (OR =  .335, 95% CI [0.118, 0.952]), and stress coping strategies of emotional support (OR = 0.533, 95% CI [0.315, 0.902]) and humor (OR = 0.648, 95% CI [0.452, 0.928]). The risk factors were: a high desire to leave the job (OR = 4.622, 95% CI [1.197, 17.847]) compared to those who do not desire to leave; perception of future health at the same level as now vs better than now (OR = 3.740, 95% CI [1.188, 11.774]); concern about infecting the family with COVID-19 (OR = 6.540, 95% CI [2.177, 19.649]) and hyperconnectivity in personal and work life (OR = 4.165, 95% CI [1.341, 12.934]), as well as greater use of stress coping strategies of planning (OR = 2.259, 95% CI [1.400, 3.644]) and self-blame (OR = 1.419, 95% CI [1.022, 1.970]) (Column 3, Table 5).

Layer 3 - by adding perception of loneliness and emotional impact of the health emergency, the model improved slightly to R^2^ = .558 (χ^2^(8) = 6.511, p = .590) and accounted for 87.6% of cases. The protective factors that entered the model were: 1) living in the capital (OR =  .345, 95% CI [0.121, 0.989]), and stress coping strategies of emotional support (OR = 0.533, 95% CI [0.314, 0.903]) and humor (OR = 0.655, 95% CI [0.456, 0.941]). The risk factors were: a high desire to leave the job (OR = 4.458, 95% CI [1.145, 17.352]) compared to those who did not have this desire; perception of future health at the same level as now vs. better than now (OR = 3.784, 95% CI [1.150, 12.450]); concern about infecting the family with COVID-19 (OR = 6.355, 95% CI [2.084, 19.380]); and hyperconnectivity in personal and work life (OR = 4.198, 95% CI [1.351, 13.050]), as well as greater use of stress coping strategies of planning (OR = 2.216, 95% CI [1.371, 3.584]), and self-blame (OR = 1.415, 95% CI [1.004, 1.996]) (Column 4, Table 5). Table 5 shows only the variables that entered the model in any of its three layers.

Finally, in order to understand the only sociodemographic factor that entered the model, a fourth-layer binary logistic regression analysis was conducted, in which the following interaction terms were included as variables: place of residence*age; place of residence*years of experience; place of residence*sex; place of residence*marital status; place of residence*ethnicity; place of residence*socio-economic level; place of residence*type of role.

**Fig 1:**
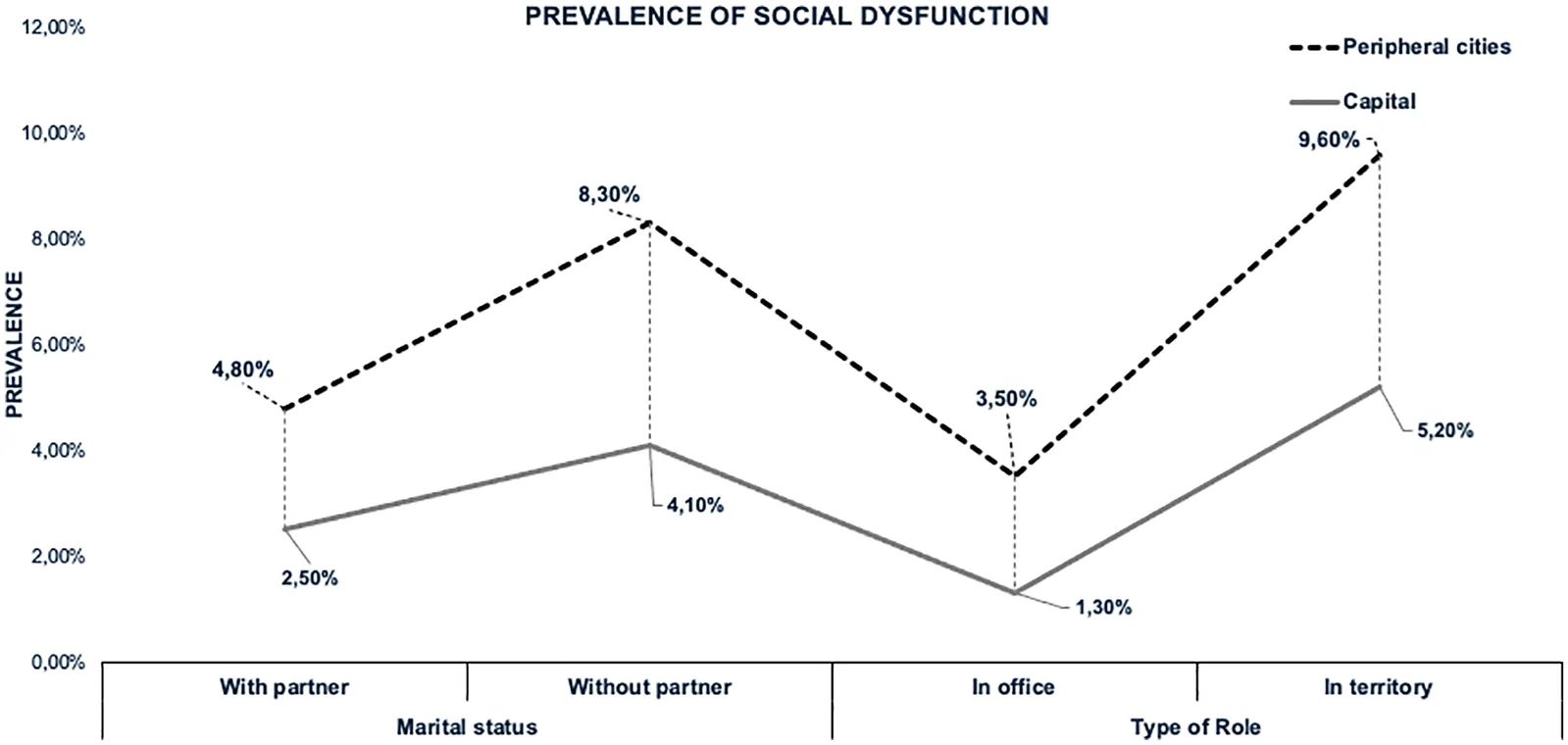
Prevalence of Social Dysfunction by Place of Residence, Marital Status and Role.

This model improved to R^2^ = .615 (χ^2^(8) = 5.386, p = .716) and accounted for 89.8% of cases. The protective factors that entered the model were: 1) living in the capital (OR =  .011, 95% CI [0.001, 0.169]), and stress coping strategies of emotional support (OR = 0.510, 95% CI [0.297, 0.875]) and humor (OR = 0.671, 95% CI [0.455, 0.988]). The risk factors were: living in peripheral cities and be mestizo (OR = 299.344, 95% CI [1.998, 44853.88]), living in peripheral cities without a formal partner (OR = 17.508, 95% CI [1.688, 181.623]), living in peripheral cities and working in field-based role (OR = 17.508, 95% CI [1.688, 181.623]), a high desire to leave the job (OR = 4.986, 95% CI [1.104, 22.523]) compared to those who did not have this desire; perception of future health at the same level as now vs. better than now (OR = 4.400, 95% CI [1.189, 16.290]); concern about infecting the family with COVID-19 (OR = 13.105, 95% CI [3.328, 51.600]); and hyperconnectivity in personal and work life (OR = 4.360, 95% CI [1.276, 14.901]), as well as greater use of planning (OR = 2.678, 95% CI [1.530, 4.687]). Fig 1 shows how the prevalence of social dysfunction is distributed according to marital status and type of role.

## Discussion

This study aimed to identify personal, occupational, and psychosocial risk and protective factors associated with social dysfunction among media workers in Ecuador, Peru, and Venezuela. First, approximately one in five journalists in the sample were at risk of social dysfunction (19.7%), and this prevalence is higher than that reported in media workers in Ecuador at the beginning of the health crisis [12], although the mean score is lower than the cut-off point, as in previous studies [10–11]. Additional analyses indicate that this risk is concentrated in specific subgroups: media workers living in peripheral cities, those identifying as individuals without a formal partner, and field-based professionals such as journalists, reporters, and photographers (see Fig 1). This pattern is consistent with evidence from peripheral Latin American cities, where journalists face greater exposure to violence, political or criminal pressure, job insecurity, and limited access to effective protection and social support systems [10,15,21,22]. By contrast, residing in Quito, Lima, or Caracas appears to be protective, likely due to the more stable working conditions and institutional support offered by major national media and other non-governmental organizations concentrated in these capitals [14].

The bivariate analysis reported an association with other sociodemographic factors: age, work experience, and female sex, but none of them acted as a risk or protective factor for social dysfunction, which contradicts what is expected based on evidence from previous research on the mental health of media workers [12,23,27]. The study also examined potential associations between place of residence and demographic variables, including age, work experience, and sex, to identify biases related to job precariousness in peripheral cities. Contrary to expectations, the results indicated that the youngest workers were concentrated in capital cities (*t*= −2.748, *p* = .006) and no significant differences were observed regarding years of experience (*t*=−1.697, *p* = .091) or sex (χ^2^ = .336, *p* = .562) when compared to those working in peripheral cities, suggesting that future studies should explore these three factors in randomly selected samples of media workers to confirm or refute these results.

The significant associations observed across sociodemographic (e.g., age, work experience, and female sex) and occupational groups (e.g., interactions between residence in peripheral cities* field-based roles; interactions between residence in peripheral cities * without a formal partner) should be interpreted as descriptive patterns rather than evidence of underlying mechanisms. Although these findings could be explained by greater exposure to stressors, or conditions of job insecurity, it is equally plausible that some other variables that have not been measured in this study may explain these findings, including broader working conditions or a baseline mental health condition. Overall, the findings contribute to identifying groups in which social dysfunction is more frequent, but do not establish why these associations occur.

The attenuation of the associations for sex, age, and work experience after the inclusion of coping strategies suggests that psychosocial characteristics account for part of the variation initially associated with these sociodemographic factors. However, given the cross-sectional design, this pattern should not be interpreted as evidence of mediation or an underlying causal mechanism. In practice, this analytical approach moves beyond the mere identification of high-risk groups to elucidate the mechanisms underlying their vulnerability, thereby allowing preventive efforts to be directed toward the most pertinent variables.

Hyperconnectivity in both personal and work domains also emerged as a risk factor. This finding is consistent with previous research linking telework demands, connectivity problems, work overload, and technostress to poorer mental health, [13,24,25,27,38], as well as with studies showing that high digital exposure and the intensive consumption and production of online information contribute to psychological distress among media workers [39]. These results underscore the need for specific regulations that guarantee media workers’ right to disconnect outside working hours [40]. Future research should further examine the differential impact of work‑related versus personal hyperconnectivity on mental health in general, and on social dysfunction in particular, and identify best practices to regulate telework and promote healthy digital content use.

In relation to occupational factors, the lack of training in crisis coverage was associated with social dysfunction, but it was not identified as a risk factor, contrary to what was expected [9,28]. The only occupational risk factor was the high desire to quit journalism, an indicator that previous studies have linked to mental health symptoms [12] and to other psychosocial risk factors frequent in the Latin American media context [24]. In this sense, future qualitative research could help deepen the understanding of the experiences of media workers who reconsider changing their life plans due to the impact of their work on their mental health.

The stressors identified as risk factors were perceiving that there is an impact on health that will not improve in the future and concern about infecting the family with COVID-19; this is consistent with what has been observed in previous studies which suggest that these factors need to be addressed in the media and in the systems for the protection of journalists [23,27,28]. The use of the self-blame stress coping strategy would also act as a risk factor for social dysfunction, consistent with what has been reported in the literature regarding passive styles [6,28,31]. This finding supports the need to investigate the experience of being a direct victim during emergencies such as the pandemic, the somatic symptoms experienced by workers, how their loved ones are affected, and how media workers cognitively process traumatic events, with self-blame possibly being one of the strategies that should be further explored through qualitative research.

The planning-based coping style was identified as a risk factor, this unexpected finding may reflect ongoing transformations in journalistic practice within an increasingly digitalized environment, characterized by constant immediacy, shorter production deadlines, and growing difficulties reported by media workers in balancing professional and family life [25,33,41]. This finding is striking, since, despite being an active coping strategy that theoretically should function as a protective factor, among media workers, who require immediate adaptation to the situation, planning does not seem to be adaptive [7,24,28,31,32]. This unexpected finding underscores the importance of investigating why media workers who tend to use planning as a coping strategy might be more vulnerable to social dysfunction.

In contrast, there are two stress coping strategies based on emotional regulation identified as protective: humor and emotional support. This is consistent with the importance of the support that can be received from others to cope with a crisis and with the need to use social skills such as humor to self-regulate and re-signify stressful events [7,24,28,31–34]. However, these associations between social support, humor, and social dysfunction should be interpreted with caution. Although greater use of emotional support and humor was associated with lower levels of social dysfunction, these findings do not establish causal relationships. One possible interpretation is that these strategies may function as protective factors. Alternatively, they may reflect underlying individual differences, such as better overall psychological functioning, which simultaneously promote adaptive coping and reduce the likelihood of social dysfunction. A further possibility is that greater use of emotional support and humor may act as mechanisms that mask an underlying need for psychological help-seeking related to distress.

Therefore, the present results do not allow us to determine whether these protective or risk factors operate as causes or consequences of social dysfunction. Future research using longitudinal or experimental designs is needed to clarify the directionality and underlying mechanisms of these associations. Also, it is important to examine whether the risk factors identified here similarly influence additional mental health outcomes among media workers. Finally, longitudinal and qualitative studies are required to explore the course and lived experiences of workers who have developed social dysfunction.

### Limitations

This study has several limitations to consider when interpreting the findings.

First, the cross-sectional design precludes any causal inference or conclusions about temporal relationships between variables. As a result, it is not possible to determine whether the identified factors act as antecedents, consequences, or correlates of social dysfunction.

Second, the use of convenience sampling limits the generalizability of the findings, as the sample may not be representative of the broader population of media workers. Individuals experiencing greater psychological distress may have been more motivated to participate, potentially inflating prevalence estimates or influencing the identification of protective factors, such as emotional support seeking.

Third, the use of screening and self-report instruments is a limitation, considering that there are currently experimental methodologies (e.g., electroencephalography, machine learning) that would allow a more objective approach [42].

Fourth, the low representation of other ethnicities in the sample suggests that the finding of the risk factor for the interaction between residing in a peripheral city and identifying as mestizo should be considered with caution.

Fifth, the study does not include direct measures of several relevant constructs discussed in the interpretation, such as working conditions or baseline mental health status. The absence of these variables limits the ability to assess alternative explanations for the observed associations.

Finally, the data were collected during two different phases of the COVID-19 pandemic, which may have influenced both perceived stressors and mental health outcomes, limiting the generalizability of the findings to non-crisis contexts.

Further research is therefore needed to deepen and corroborate these findings in other crisis scenarios.

## Conclusion

In conclusion, the prevalence of social dysfunction in this sample of media workers in Ecuador, Peru and Venezuela is 19.7%. The only sociodemographic factor associated with a reduced risk of social dysfunction is residing in capital cities, and the highest prevalences are observed among single and field-based workers in the peripheral cities of Ecuador, Peru, and Venezuela. Work-related factors that increase the risk of social dysfunction are the desire to change jobs, the perception that health will not improve, the fear of infecting the family and hyperconnectivity. Finally, the stress coping strategies that increase the risk of social dysfunction are planning and self-blame, while those that reduce risk are humor and seeking emotional support.

## Supporting information

S1 Database (SAV)

## References

[1] Shafer A. Meta-analysis of factor analyses of the general health questionnaire – short forms GHQ-28 and GHQ-30. European J Psychological Assessment. 2024;40(1):46–58. doi: 10.1027/1015-5759/a000727

[2] Lobo A, Pérez-Echeverría MJ, Artal J. Validity of the scaled version of the General Health Questionnaire (GHQ-28) in a Spanish population. Psychol Med. 1986;16(1):135–40. doi: 10.1017/s0033291700002579 3961039

[3] Bagheri S, Taridashti S, Farahani H, Watson P, Rezvani E. Multilayer perceptron modeling for social dysfunction prediction based on general health factors in an Iranian women sample. Front Psychiatry. 2023;14:1283095. doi: 10.3389/fpsyt.2023.1283095 38161726 PMC10756140

[4] Aoki Y, Malcolm E, Yamaguchi S, Thornicroft G, Henderson C. Mental illness among journalists: a systematic review. Int J Soc Psychiatry. 2013;59(4):377–90. doi: 10.1177/0020764012437676 22408118

[5] MacDonald JB, Saliba AJ, Hodgins G. Journalists and substance use: A systematic literature review. Subst Abus. 2016;37(3):402–11. doi: 10.1080/08897077.2015.1101732 26453337

[6] MacDonald JB, Saliba AJ, Hodgins G, Ovington LA. Burnout in journalists: A systematic literature review. Burnout Research. 2016;3(2):34–44. doi: 10.1016/j.burn.2016.03.001

[7] MacDonald JB, Hodgins G, Saliba AJ, Metcalf DA. Journalists and Depressive Symptoms: A Systematic Literature Review. Trauma Violence Abuse. 2023;24(1):86–96. doi: 10.1177/15248380211016022 33998329

[8] Feinstein A, Wanga J, Owen J. The psychological effects of reporting extreme violence: a study of Kenyan journalists. JRSM Open. 2015;6(9):2054270415602828. doi: 10.1177/2054270415602828 26464808 PMC4589076

[9] McMahon C. Covering disaster: A pilot study into secondary trauma for print media journalists reporting on disaster. Australian Journal of Emergency Management. 2001;16:52–6.

[10] Feinstein A. Mexican journalists and journalists covering war: a comparison of psychological wellbeing. Journal of Aggression, Conflict and Peace Research. 2013;5(2):77–85. doi: 10.1108/17596591311313672

[11] Feinstein A, Starr S. Civil War in Syria: the psychological effects on journalists. Journal of Aggression, Conflict and Peace Research. 2015;7(1):57–64. doi: 10.1108/jacpr-04-2014-0119

[12] Bustamante-Granda BF, Rodríguez-Hidalgo C, Cisneros-Vidal MA, Rivera-Rogel D, Torres-Montesinos C. Ecuadorian Journalists Mental Health Influence on Changing Job Desire: A Cross Sectional Study. Int J Environ Res Public Health. 2021;18(19):10139. doi: 10.3390/ijerph181910139 34639441 PMC8508482

[13] Dunwoody S. Science Journalism and Pandemic Uncertainty. Media Communication. 2020;8(2):471–4. doi: 10.17645/mac.v8i2.3224

[14] Vázquez Valdez JA. Periodismo y criminalidad, relación que se estrecha bajo dinámicas neoliberales y de dominación. Ámbitos Revista Internacional de Comunicación. 2022;(56):164–77.

[15] Johamna-María Muñoz-Lalinde, Julián Jiménez-Madiedo, Pedro Molina-Rodríguez-Navas. Censorship and self-censorship of Colombian journalists when addressing information related to local administrations. Profesional de la información. 2024;33(5). doi: 10.3145/epi.2024.0502

[16] Maciaszek J, Ciulkowicz M, Misiak B, Szczesniak D, Luc D, Wieczorek T, et al. Mental Health of Medical and Non-Medical Professionals during the Peak of the COVID-19 Pandemic: A Cross-Sectional Nationwide Study. J Clin Med. 2020;9(8):2527. doi: 10.3390/jcm9082527 32764509 PMC7463597

[17] Teng Z, Wei Z, Qiu Y, Tan Y, Chen J, Tang H, et al. Psychological status and fatigue of frontline staff two months after the COVID-19 pandemic outbreak in China: A cross-sectional study. J Affect Disord. 2020;275:247–52. doi: 10.1016/j.jad.2020.06.032 32734915 PMC7330556

[18] Feinstein A, Owen J, Blair N. A hazardous profession: war, journalists, and psychopathology. Am J Psychiatry. 2002;159(9):1570–5. doi: 10.1176/appi.ajp.159.9.1570 12202279

[19] Monteiro S, Marques Pinto A, Roberto MS. Job demands, coping, and impacts of occupational stress among journalists: a systematic review. European Journal of Work and Organizational Psychology. 2015;25(5):751–72. doi: 10.1080/1359432x.2015.1114470

[20] Gutiérrez-Coba LM. Situación profesional y satisfacción laboral de los periodistas colombianos. Comysoc. 2020;:1–26. doi: 10.32870/cys.v2020.7556

[21] González Macías RA, Cepeda Robledo DA. Trabajar por amor al arte: precariedad laboral como forma de violencia contra los periodistas en México. Global Media Journal México. 2021;18(34):209–28. doi: 10.29105/gmjmx18.34-10

[22] Valencia Espinoza G. Acentos y silencios en la protección a periodistas en Ecuador. Revista Enfoques de la Comunicación. 2020;(3):182–215.

[23] Gottfried J, Mitchell A, Jurkowitz M, Liedke J. Older, younger journalists differ in views of work, use of social media. Pew Research Center. 2022. https://www.pewresearch.org/journalism/2022/06/14/older-journalists-most-content-in-their-work-younger-journalists-more-likely-to-find-social-media-helpful/

[24] Bustamante Granda BF, Torres-Montesinos C, Rivera-Rogel D, Rodríguez-Hidalgo C, Villar F. Factores psicosociales asociados al deseo de dejar de trabajar en medios de comunicación de Ecuador, Perú y Venezuela. Revista de Comunicación. 2025;23(1):95–108. doi: 10.26441/RC24.1-2025-3697

[25] de Luis Andrés M. Reporting under pressure a study of journalism working conditions in three European countries during the pandemic. Sundsvall / Östersund: Research centre/department Media and Communication Studies / DEMICOM. 2022.

[26] Miller KC. Harassment’s Toll on Democracy: The Effects of Harassment Towards US Journalists. Journalism Practice. 2021;17(8):1607–26. doi: 10.1080/17512786.2021.2008809

[27] Miret M. La salud mental de los periodistas se resiente por los efectos de la pandemia. Cuadernos de Periodista. 2021; 42:31–43. Available from: https://dialnet.unirioja.es/servlet/articulo?codigo=7981899

[28] Bustamante B, Carrión G, Velázquez A. Estilos de afrontamiento al estrés por Covid-19 en periodistas ecuatorianos. In: Cuesta U, Barrientos A, Martínez L, editors. Los nuevos materiales de comunicación y salud. 1a ed. Madrid: Fragua. 2022. p. 341–56.

[29] Tejedor S, Cervi L, Tusa F. Periodismo en contextos de violencia, principales problemas y posibles vías de solución: percepciones de periodistas latinoamericanos. Revista de Comunicación. 2022;21(2):285–306. doi: 10.26441/RC21.2-2022-A14

[30] Kola L, Kohrt BA, Hanlon C, Naslund JA, Sikander S, Balaji M, et al. COVID-19 mental health impact and responses in low-income and middle-income countries: reimagining global mental health. Lancet Psychiatry. 2021;8(6):535–50. doi: 10.1016/S2215-0366(21)00025-0 33639109 PMC9764935

[31] Wang L, Kang C, Yin Z, Su F. Psychological endurance, anxiety, and coping style among journalists engaged in emergency events: evidence from China. Iran J Public Health. 2019;48(1):95–102. doi: 10.18502/ijph.v48i1.787 30847316 PMC6401570

[32] Beam RA, Spratt M. Managing vulnerability. Journalism Practice. 2009;3(4):421–38. doi: 10.1080/17512780902798653

[33] Backholm K, Idås T. Journalists and the coronavirus. How changes in work environment affected psychological health during the pandemic. Journalism Practice. 2022;18(6):1560–76. doi: 10.1080/17512786.2022.2098522

[34] Idås T, Backholm K, Korhonen J. Trauma in the newsroom: social support, post-traumatic stress and post-traumatic growth among journalists working with terror. Eur J Psychotraumatol. 2019;10(1):1620085. doi: 10.1080/20008198.2019.1620085 31231480 PMC6566646

[35] Velarde-Mayol C, Fragua Gil MS, García de Cecilia JM. Validación de la escala de soledad de UCLA y perfil social en la población anciana que vive sola. Semergen: revista española de medicina de familia. 2016;(3):177–83. 10.1016/j.semerg.2015.05.017 26187595

[36] Morán Astorga C, Landero Hernández R, González Ramírez MT. COPE-28: un análisis psicométrico de la versión en español del brief COPE. Univ Psychol. 2010;9(2):543–52. doi: 10.11144/javeriana.upsy9-2.capv

[37] Arias PR. Escala breve de gravedad del estrés por la percepción de la emergencia sanitaria COVID -19. NeuroCog. 2020. doi: 10.13140/RG.2.2.33061.24809

[38] Bernadas JMAC, Ilagan K. Journalism, public health, and COVID-19: some preliminary insights from the Philippines. Media International Australia. 2020;177(1):132–8. doi: 10.1177/1329878X20953854

[39] Bozovic I, Jovic-Vranes A, Stasevic-Karlicic I, Stanisavljevic D, Pavlovic V, Todorovic J. Exploring the association between digital health literacy and burnout and depression among TV journalists during the COVID-19 Pandemic in Serbia. Healthcare (Basel). 2025;13(14):1688. doi: 10.3390/healthcare13141688 40724713 PMC12295177

[40] James-Garrod C. Disconnection, distraction, and time deficits: how Australian journalists report smartphones affect their work-related duties. Communication Research and Practice. 2025;11(2):205–20. doi: 10.1080/22041451.2025.2491281

[41] Deuze M. Considering mental health and well-being in media work. Australian Journalism Review. 2023;45(1):15–26. doi: 10.1386/ajr_00115_7

[42] Yadulla AR, Sajja GS, Addula SR, Maturi MH, Nadella GS, De La Cruz E, et al. A systematic review of mental health monitoring and intervention using unsupervised deep learning on EEG data. Psychology International. 2025;7(3):61. doi: 10.3390/psycholint7030061

