# Supplementary material for: Social dysfunction in Ecuadorian, Peruvian, and Venezuelan media workers
Source: PLOS Ment Health. 2026 Sep 2;3(9):e0000709. doi: 10.1371/journal.pmen.0000709 (PMC13537591; doi:10.1371/journal.pmen.0000709)
Supplement: Supplementary file 3 [file pmen.0000709.s004.docx]

Dear Editor and Reviewer #3,

We would like to sincerely thank the Editor and Reviewer #3 for the careful evaluation of our manuscript and for the positive and constructive comments provided throughout the review process.

We are grateful for the reviewer’s assessment that the manuscript satisfactorily addresses the concerns raised during the previous review rounds and that it meets the publication criteria of *PLOS Mental Health*. We appreciate the recognition of the improvements made in the clarity of presentation, methodological description, organization of the manuscript, and interpretation of findings.

In response to the reviewer’s recommendation regarding the final language review, we conducted a comprehensive revision of the entire manuscript. Specifically, we:

- Improved grammatical accuracy throughout the text.
- Corrected minor spelling and punctuation issues.
- Refined the wording of several sentences to enhance clarity and readability.
- Reviewed and standardized terminology to ensure consistency across all sections of the manuscript.
- Performed a final academic English language and style review.

All modifications have been clearly identified in the file **“Revised Manuscript with Track Changes.”**

Additionally, we have included **Mgtr. David Fernando Jácome** and **Mgtr. Galo Ojeda** in the **Acknowledgments** section in recognition of their valuable contribution to the linguistic and stylistic revision of the final version of the manuscript.

We thank the Editor and Reviewer #3 for their time, constructive feedback, and positive evaluation of our work. We believe that the revisions have further improved the quality and clarity of the manuscript, and we respectfully submit the revised version for your consideration.

Sincerely,

**Byron Fernando Bustamante Granda**

Corresponding Author

On behalf of all co-authors

**Response to Reviewer #3**

**Comment:**
*The English is generally sufficient to communicate the scientific content; however, a few minor issues related to grammar, wording, and terminological consistency remain and should be corrected through a final language review before publication.*

**Response:**
Thank you for this valuable observation. Following your recommendation, we conducted a thorough linguistic and stylistic review of the entire manuscript. Grammar, spelling, punctuation, wording, and terminological consistency were carefully revised to improve clarity and readability in academic English. All changes have been highlighted in the version submitted with tracked changes. Furthermore, we acknowledged **Mgtr. David Fernando Jácome** and **Mgtr. Galo Ojeda Brito** for their contribution to the final linguistic and style review of the manuscript.

**Action Taken:**

A comprehensive English language and style revision was completed throughout the manuscript, and all modifications are visible in the tracked-changes version.
