# Supplementary material for: Social dysfunction in Ecuadorian, Peruvian, and Venezuelan media workers
Source: PLOS Ment Health. 2026 Sep 2;3(9):e0000709. doi: 10.1371/journal.pmen.0000709 (PMC13537591; doi:10.1371/journal.pmen.0000709)
Supplement: Supplementary file 4 [file pmen.0000709.s002.docx]

Response to Reviewers - Manuscript PMEN-D-26-00016

**Title:** Social dysfunction in Ecuadorian, Peruvian, and Venezuelan media workers

**Dear Dr. Martin Mabunda Baluku, Ph.D. and Editorial Board,**

**PLOS Mental Health**

Thank you for the opportunity to revise our manuscript. We appreciate the constructive feedback provided by you and the reviewers, which has significantly helped us improve the clarity and depth of our study.

We have addressed all concerns raised, specifically regarding the financial disclosure, the statistical interpretation of the regression layers, and the linguistic clarity of the text.

Below, we provide a point-by-point response to the comments.

We have updated the Financial Disclosure statement as requested. It now reads:

*This study received funding from the United Nations Educational, Scientific and Cultural Organization UNESCO through Grant No. 314565, Montevideo office (Uruguay), under contract No. 4500447751-A1 (to BB and CT). It was co-financed by the Universidad Técnica Particular de Loja, through the project Mental Health in Latin American Journalists during the COVID-19 pandemic (Code: PY3222) (to BB, CT, and JE). The funders had no role in study design, data collection and analysis, decision to publish, or preparation of the manuscript. The Universidad de Las Américas (to BB) and the Universidad Técnica Particular de Loja (to CT and JE) contributed to the salaries of the three researchers in this article as co-financing.*

**Reviewer #1**

**Observation 1. Using a convenience sample may limit how well the findings represent the entire media population in these countries.**

Response 1: We concur with the reviewer’s assessment regarding the methodological constraints of convenience sampling. We acknowledge that this approach may limit the external validity and generalizability of our findings to the broader media population in Ecuador, Peru, and Venezuela. To address this, we have expanded the "Limitations" section to explicitly discuss the potential for selection bias and its impact on the reported 19.7% prevalence rate. Consequently, the interpretation of our results and the conclusions have been refined to emphasize that these findings should be viewed as indicative of the studied cohort rather than a definitive extrapolation to all media workers in the region.

**Note 1: See lines 426-435 in the "Discussion" and "Limitations" sections, and lines 444 anddirection 445 in the conclusion.**

**Observation 2. The study mentions "hyperconnectivity" as a risk factor but does not analyze how different digital platforms (like social media compared to traditional broadcasting) might affect this risk.**

We appreciate this insightful observation. We have clarified that the "hyperconnectivity" construct evaluated in this study was conceptualized as a holistic measure of aggregate digital exposure, spanning both personal and professional spheres. The primary objective was to assess the perceived overall level of digital connection rather than to differentiate the specific impact of various platforms (e.g., social media vs. traditional broadcasting) or to isolate work-related versus leisure-related usage.

To ensure conceptual and methodological clarity, we have standardized the use of the term "**hyperconnectivity in personal and work life**" throughout the manuscript. This refinement explicitly delimits the scope of the measured factor and prevents misinterpretations regarding platform-specific analysis. Furthermore, we have expanded the "Discussion" section to highlight the necessity for future research to utilize more granular metrics to disentangle the distinct risk mechanisms associated with different digital environments. Two pertinent references (39 and 40) have been added to support this future direction.

**Note 2: See lines 362-364 and references 39 and 40**

**Observation 3. The manuscript assumes that social dysfunction does not have cases of "spontaneous recovery." However, it does not provide long-term data to support this claim for this specific group.**

We appreciate the reviewer's critical insight regarding the assumption of spontaneous non-remission. We have corrected this by removing the definitive claim throughout the manuscript. Instead, we now state that empirical evidence, such as the longitudinal study by McMahon et al. [9] on Australian journalists, suggests that while symptoms of anxiety and insomnia may fluctuate, levels of social dysfunction tend to remain relatively stable when comparing the period of trauma exposure to the journalists' current condition.

Furthermore, we have clarified that although our study is not longitudinal in design, it employs a retrospective approach to explore the pre- and post-traumatic experiences of the participants. Consequently, we have rephrased our interpretations to avoid assuming a lack of spontaneous recovery, presenting the observed stability of social dysfunction as a specific characteristic of this cohort that warrants further longitudinal investigation.

**Note 3: See lines 90 -92**

**Observation 4. Some parts of the Results and Discussion should connect more clearly. For example, correlating the bivariate results with the final regression model could explain why certain demographic factors were excluded.**

Response 4: We appreciate this suggestion, which has allowed us to significantly strengthen the coherence between our Results and Discussion sections. We have implemented the following structural and analytical clarifications:

Initially, bivariate analyses indicated associations between social dysfunction and variables such as age, sex, and years of experience. However, when these were entered into the binary logistic regression model, both as main effects and through interactions with "place of residence", they did not yield substantive improvements in model fit or explanatory power (contrary to theoretical expectations).

In contrast, the inclusion of the final layer (Layer 4), which incorporated interactions between "place of residence" and "ethnicity," "professional role," and "marital status," significantly enhanced the model’s predictive accuracy from **87.6% to 89.8%**. This refined model demonstrates that psychosocial and contextual interactions are more robust predictors of social dysfunction than isolated demographic factors.

Specifically, the final model identifies:

1. Stable Protective Factors: Residing in the capital, use of humor, and emotional support.
2. Emergent Risk Factors (Interactions): Living in a peripheral city while being single/without a partner, identifying as mestizo, or performing field-based work.
3. Adjusted Factors: A reduction in the weight of "self-blame" as a risk factor, while maintaining the significance of "future health perception," "desire to change jobs," "fear of infecting family," "hyperconnectivity," and "use of planning."

By prioritizing these interactions, the analysis shifts focus from non-modifiable variables (sex, age) toward modifiable contextual and individual factors, thereby increasing the manuscript’s relevance for practical mental health interventions.

**Note 4: See Figure 1, lines 303-317.**

**Observation 5. There should be a more thorough discussion of the limitations of convenience sampling and how it might have affected the reported 19.7% prevalence rate.**

Response 5: We agree that the use of convenience sampling necessitates a more nuanced interpretation of the reported prevalence rate. In response to this observation, we have significantly expanded the "Limitations" section to analyze how selection bias may have influenced the 19.7% prevalence of social dysfunction.

Specifically, we discuss the possibility that individuals experiencing higher levels of distress might have been more motivated to participate, potentially overestimating the prevalence. And propose the use of random sampling in future projects.

**Note 5: See lines 344-345 and 426 and 430**

**Observation 6. In the discussion about the need for objective, scalable tools for monitoring mental health. It is suggested to refer to the paper: “Mental Health Monitoring and Intervention Using Unsupervised Deep Learning on EEG Data”. This reference is important because it highlights the urgent need for objective, scalable, and accessible tools for early detection of mental health issues like depression and anxiety. While this manuscript uses self-report scales (GHQ-28), discussing advanced monitoring techniques such as EEG data analysis would strengthen the foundation by showing future directions for mental health monitoring beyond surveys. This would help connect traditional screening methods with emerging objective tools.**

Response 6: We appreciate the reviewer's excellent recommendation. We have integrated the suggested study into the manuscript to strengthen our discussion on the evolution of mental health assessment. Specifically, we have utilized this reference to contrast the limitations of traditional self-report instruments (such as the GHQ-28) with emerging objective, scalable, and accessible diagnostic technologies.

By citing this work, we highlight the urgent need for a transition toward experimental and automated methodologies, such as unsupervised deep learning applied to EEG data, for the early detection of anxiety and depression. This addition not only provides a more robust theoretical foundation for our research but also positions our findings within a broader, forward looking context regarding the future of mental health monitoring in high stress occupational environments.

**Note 6: See lines 430-433 and reference 42.**

**Observation 7. The assumption about the lack of spontaneous recovery needs to include references to existing long-term studies using the GHQ-28 Scale C or should state this as a limitation for future research.**

Response 7: We appreciate this observation. The statement regarding the absence of spontaneous recovery was due to an incorrect interpretation of one of the previously cited studies, so it has been corrected in the manuscript. This aspect was corrected in response to Observation 3. However, the reviewer's suggestion is maintained by incorporating into the discussion the need to develop future longitudinal research to explore the course of social dysfunction in journalists.

**Note 7: See lines 90- 93 and 439**

**Observation 8. The Results section should include a clearer explanation of the shift between Layer 1 and Layer 3 of the regression model. This will clarify why initial risk factors like "sex" and "age" were replaced by psychosocial factors.**

Response 8: We appreciate this observation, which allowed us to clarify the transition from our preliminary analyses to the Layered Binary Logistic Regression. We have updated both the Results and Discussion sections to provide a more transparent explanation of this process.

While preliminary bivariate analyses showed significant associations between social dysfunction and sociodemographic factors (such as sex, age, and years of experience), these variables did not maintain statistical significance once entered into the initial multivariate model (Layer 1). This suggests that while these biographical characteristics may appear relevant in isolation, their individual predictive power is negligible when considered alongside other structural and contextual factors.

As the model progressed to Layer 2 and Layer 3, the inclusion of psychosocial variables, specifically coping strategies, further demonstrated that these are the proximal explanatory mechanisms of social dysfunction. In this framework, biographical characteristics operate as distal variables whose potential influence is effectively subsumed by more robust psychosocial and contextual predictors. This indicates that the risk of social dysfunction is driven more by the differential distribution of psychosocial resources and specific working conditions than by inherent demographic traits.

Consequently, we have refined the Discussion to explain that the displacement of these variables in the final model highlights the importance of focusing on modifiable intervention points rather than non-modifiable demographic factors.

**Note 8: See lines 346-354**

**Observation 9.** The language in the Introduction should be refined to use more precise terms when discussing "trauma repair processes."

Response 9: We acknowledge the reviewer’s observation regarding the lack of conceptual precision in the term "trauma repair processes." We agree that this phrasing did not accurately reflect the established psychological literature. Consequently, we have revised the introduction to utilize more rigorous terminology.

**Note 9: See line 69**

**Observation 10. All references within the text must strictly follow the journal's citation style, especially regarding the brackets used in the reference list.**

Response 10: We appreciate the reviewer’s attention to detail. A comprehensive audit of the manuscript’s referencing system has been conducted. We have ensured that all in-text citations and the final reference list strictly adhere to the PLOS Mental Health style guidelines, with particular emphasis on the correct implementation of square brackets [] and the standardization of bibliographic metadata. All formatting inconsistencies have been rectified to meet the journal's formal requirements.

**Reviewer #2**

**Observation 1. The manuscript needs editing to remove section that spontaneously to Spanish and other sections that are simply not clear.**

Response 1: We sincerely apologize for the presence of inadvertent Spanish fragments and for the lack of clarity in certain passages. To address these issues, the manuscript has undergone a comprehensive linguistic and stylistic revision.

All non-English text has been eliminated, and a rigorous proofreading process was conducted to ensure terminological consistency, conceptual precision, and fluency in academic English. Furthermore, we have reformulated ambiguous phrasing throughout the document to enhance expository clarity and ensure that our findings are presented in an intelligible fashion, meeting the high standards of PLOS Mental Health.

**Observation 2. The paper does not account for the fact that younger, early-career journalists are more likely to take positions in rural or peripheral regions because these roles are less desirable, lower paid, higher risk, but offer entry into the profession and initial bylines. This factor may account for the bulk of your findings and not be easy to change.**

Response 2.

We are particularly grateful for this insightful observation, as it addresses a fundamental structural dynamic of the journalistic labor market. We concur that the tendency for early-career journalists to occupy high-risk, low-pay positions in peripheral regions as an entry point into the profession constitutes a plausible explanatory framework for professional distress and social dysfunction.

To address this, we integrated additional analyses examining the intersections between **place of residence, age, sex, and professional experience**. Interestingly, our data revealed that in this specific cohort, younger workers were more frequently concentrated in capital cities, and no significant association was found between place of residence and years of experience or gender.

Furthermore, as noted in our response to Reviewer 1 (Observation 8), while these sociodemographic factors appeared relevant in preliminary bivariate analyses, they lost statistical significance in the logistic regression once psychosocial variables and coping strategies were introduced. This reinforces the interpretation that sex, age, and experience operate as distal variables, whereas coping mechanisms act as proximal explanatory drivers of social dysfunction.

Consequently, our findings suggest that the observed social dysfunction is not merely an artifact of early labor insertion in peripheral areas, but rather a reflection of territorial inequalities in access to support resources. By identifying the specific interactions between territory, professional role, and marital status, our model highlights modifiable factors that are critical for targeted mental health interventions in the Latin American context.

**Note 10. See Figure 1, Lines 63-65, 303-317, 334-345**

Finally, the manuscript has been meticulously revised to provide a more precise occupational taxonomy of the professional roles within the media sector. We have enhanced the contextual framing of the analyzed work environments to facilitate a more robust interpretation of the findings and their practical implications. Specifically, the "Participants" section now offers a more granular description of the diverse roles and types of media organizations involved, ensuring a clearer understanding of the specific labor conditions and professional hierarchies inherent to this cohort.

**Note 11. See lines 137-142**

We are sincerely grateful for the constructive feedback provided by the reviewers. We believe that addressing these observations and incorporating the additional analyses has substantially strengthened the manuscript's rigor and clarity. Our findings provide critical evidence to guide future mental health prevention strategies addressing social dysfunction among media workers from a necessary territorial perspective. This study provides rare and essential empirical evidence on the psychological well-being of 'second-line' professionals in developing countries, bridging a critical gap for future mental health policy.
