# Supplementary material for: Social dysfunction in Ecuadorian, Peruvian, and Venezuelan media workers
Source: PLOS Ment Health. 2026 Sep 2;3(9):e0000709. doi: 10.1371/journal.pmen.0000709 (PMC13537591; doi:10.1371/journal.pmen.0000709)
Supplement: Supplementary file 5 [file pmen.0000709.s003.docx]

Response to Reviewers - Manuscript PMEN-D-26-00016R1

**Title:** Social dysfunction in Ecuadorian, Peruvian, and Venezuelan media workers

**Dear Dr. Martin Mabunda Baluku, Ph.D. and Editorial Board,**

**PLOS Mental Health**

Dear Editor and Reviewers, we would like to express our sincere gratitude for the time and effort you have dedicated to reviewing our manuscript, as well as for your insightful and constructive comments.

In the table below, we present a detailed, point-by-point response to each reviewer comment, outlining the revisions made and indicating the corresponding line numbers in the final version of the manuscript

**Editor**

**Observation 1: Ensure that the recommendations are not overstated and are based on the findings.**

**Response 1:** We thank the Editor for this valuable observation. In response, we have thoroughly revised the manuscript to ensure that all recommendations are closely aligned with the study’s findings and are not overstated. Specifically, we have made substantial revisions to the Discussion section, moderating the language to avoid causal interpretations and ensuring that all implications are clearly grounded in the empirical results. Additionally, the recommendations have been reframed to focus on directions for future research, aiming to deepen the understanding of the study’s findings rather than proposing applied implications. We have also emphasized the exploratory nature of the study and the need for further research before broader generalizations are warranted. We greatly appreciate this guidance, as it has contributed to improving the accuracy and balance of our interpretations.

**Reviewer #1**

**General Observation: I recommend accepting the manuscript. The authors have thoroughly revised it, addressing all major reviewer comments and making necessary modifications. The revised version now meets the journal’s standards for quality, clarity, and ethics, and is ready for publication, offering a valuable contribution to the field.**

**1. The authors responded clearly and made major changes, refining the "hyperconnectivity" idea and fixing assumptions about spontaneous recovery.**

**2. The manuscript was carefully revised to improve language, remove non-English parts, and connect theory with results.**

**3. Layered binary logistic regression clarifies how distant social factors relate to psychological mechanisms.**

**4. This study offers vital evidence on social issues affecting underrepresented media workers in Latin America, filling a gap in global mental health research.**

**5. Adding interaction terms in Layer 4 increased model accuracy to 89.8%, giving clearer risk factor insights in urban areas.**

**General Response:** We sincerely thank the reviewer for the positive and thoughtful evaluation of our manuscript.

We greatly appreciate the recognition of the revisions undertaken, particularly regarding the clarification of the hyperconnectivity construct, the refinement of theoretical assumptions, and the improvements in language and overall coherence.

We are also grateful for the acknowledgment of the contribution of our analytical approach, including the hierarchical binary logistic regression model and the incorporation of interaction terms, which strengthened the interpretation of psychosocial and contextual factors.

Finally, we value the reviewer’s recognition of the relevance of this study in addressing underrepresented populations within global mental health research. We are pleased that the revised manuscript meets the journal’s standards and is considered a valuable contribution to the field.

**Reviewer #2**

**Observation 1: In this manuscript, the authors report on a survey of social dysfunction among media workers. Although the manuscript is more readable than earlier versions, with the prior problems of switching into Spanish and other unclear passages apparently corrected, the central interpretive weaknesses remain unresolved.**

**Response 1:** We sincerely thank the reviewer for the careful re-evaluation of the manuscript and for acknowledging the improvements in readability and clarity achieved in this revised version. We also appreciate the continued critical perspective regarding the central interpretation of the findings.

In response to this concern, we have further refined the manuscript to strengthen its interpretative framework, aiming to provide a more robust and theoretically grounded interpretation of the results, avoiding overgeneralizations and ensuring that the conclusions are fully supported by the data.

We are grateful for this observation, which has contributed to improving the conceptual rigor and interpretative clarity of the manuscript.

**Observation 2: The main problem is that the data do not support the recommendations. The manuscript describes correlates of social dysfunction, but it does not establish the mechanisms underlying it, nor does it provide evidence linking the observed associations to the proposed interventions.**

**Response 2.1:**

We sincerely thank the reviewer for this critical and valuable observation. We acknowledge that, in the previous version of the manuscript, the recommendations extended beyond what could be directly supported by the study design and data.

In response, we have substantially revised the manuscript to ensure full alignment between the study’s objectives, analytical scope, and conclusions. Specifically, we have clarified in the Discussion section that the primary objective of this study is to identify risk and protective factors associated with social dysfunction, rather than to establish causal mechanisms or derive direct intervention strategies (lines 321 to 323).

Consistent with the reviewer’s comment, we recognize that the cross-sectional design and correlational nature of the analyses do not provide sufficient evidence to support mechanistic explanations or to justify prescriptive recommendations. Therefore, we have removed the previously included list of practical recommendations, which exceeded the empirical scope of the study.

Additionally, we have revised the Discussion to provide a clearer conceptual thread, ensuring that interpretations remain grounded in the observed associations and that the exploratory nature of the findings is explicitly acknowledged. In line with this, we have also strengthened the Limitations section by explicitly stating: “***First, the cross-sectional design precludes any causal inference or conclusions about temporal relationships between variables. As a result, it is not possible to determine whether the identified factors act as antecedents, consequences, or correlates of social dysfunction***” (lines 433 to 436).

Following the reviewer’s observation, we have also redirected our approach away from prescriptive intervention recommendations and instead framed our contributions in terms of future research directions. Specifically, in relation to hyperconnectivity, we have incorporated the following statement in the Discussion (lines 375 to 378):

“***Future research should further examine the differential impact of work‑related versus personal hyperconnectivity on mental health in general, and on social dysfunction in particular, and identify best practices to regulate telework and promote healthy digital content use****.”*

This adjustment ensures that our conclusions remain aligned with the scope of our findings, avoiding overinterpretation while still highlighting relevant avenues for future investigation.

**Observation 2 (continue): The authors report greater social dysfunction among younger participants, those with less training, poorer health, loneliness, self-blame, and concerns about their own health and about infecting family members. Presumably, this concern relates to COVID-19, but the timing of the survey in relation to the pandemic is not clearly stated and should be specified.**

**Response 2.2:** we have revised the manuscript to explicitly clarify the temporal context of the study in relation to the COVID-19 pandemic. Specifically, this information has been incorporated into multiple sections of the manuscript to ensure clarity and consistency: in the Abstract (lines 26,31), the Methods section (line 160), the Results section (lines 239, 264, 275, 288, 313) and the discussion section (line 388), as well as in Tables 3 and 5, where the pandemic context is now explicitly stated.

Additionally, the Discussion section has been expanded to further elaborate on the interpretation of these findings, particularly regarding concerns about personal health and the risk of transmitting infection to family members. In this context, we also highlight the need for future research to more directly examine the perceived personal and family-related impact of exposure to emergency conditions among media workers.

These revisions ensure that the relationship between the reported concerns and the COVID-19 context is clearly specified (lines 393 to 398).

**Observation 2 (continue): Lower risk was observed among those living in capital cities and among those seeking emotional support or using humor as a coping strategy. However, these findings remain descriptive. They do not explain why social dysfunction occurs, nor do they identify a supported target for intervention.
A major interpretive flaw is the suggestion that limited access to social support explains the social dysfunction observed in 19.7% of the 314 individuals studied. The data do not demonstrate this. There is no evidence here that lack of social support is a causal factor. Variables such as loneliness, reduced help-seeking, and lower use of humor may simply reflect broader psychological capability or poorer overall mental health, mechanisms that are correlated with social dysfunction. The manuscript does not disentangle these possibilities. The manuscript offers interventions, but provides no data indicating that these recommendations are likely to be effective.**

**Response 2.3:** We thank the reviewer for these important observations regarding the interpretative scope of our findings. We agree that the original version of the manuscript did not sufficiently disentangle alternative explanations for the observed associations.

In response to the concern that the reported associations remain descriptive and do not explain underlying mechanisms, we have revised the manuscript to explicitly acknowledge this limitation. A clarifying paragraph has been incorporated in the Discussion (lines 349 to 358), stating:

***“The significant associations observed across sociodemographic (e.g. age, work experience, and female sex) and occupational groups (e.g. interactions between residence in peripheral cities* field-based roles; interactions between residence in peripheral cities * without a formal partner) should be interpreted as descriptive patterns rather than evidence of underlying mechanisms. Although these findings could be explained by greater exposure to stressors, or conditions of job insecurity, it is equally plausible that some other variables that have not been measured in this study may explain these findings, including broader working conditions or a baseline mental health condition. Therefore, the findings contribute to identifying groups in which social dysfunction is more frequent, but do not establish why these associations occur.”***

This addition ensures that the manuscript clearly distinguishes between correlational findings and mechanistic explanations, aligning the interpretation with the study design.

Regarding the concern about the interpretation of social support and coping strategies, we acknowledge that the previous version of the manuscript overstated the role of limited access to social support as an explanatory factor. In response, we have removed statements implying causal relationships not supported by the data. We have also refined the interpretation of variables such as humor and emotional support. These variables are now presented strictly as associations, without implying causal mechanisms or direct intervention targets. We explicitly acknowledge that they may reflect broader psychological functioning or overall mental health status, rather than acting as independent explanatory drivers of social dysfunction (lines 413 to 422).

Consistent with this revision, and as noted in our previous responses, we have removed prescriptive intervention recommendations that were not sufficiently supported by the data. This ensures a clear distinction between empirical findings and their potential implications.

In addition, we have strengthened the Limitations section to explicitly acknowledge potential biases related to the detection of protective factors. Specifically, we note that the use of convenience sampling may have influenced not only prevalence estimates but also the identification of associations such as emotional support, as individuals experiencing greater psychological distress may have been more motivated to participate (lines 437 to 441).

Finally, we have incorporated an additional limitation acknowledging that the study does not include direct measures of several relevant constructs referenced in the interpretation, such as working conditions and baseline mental health status. The absence of these variables limits our ability to evaluate alternative explanations and further supports interpreting the findings as correlational rather than explanatory (lines 448 to 451).

These modifications strengthen the conceptual rigor of the study and ensure that all interpretations remain appropriately grounded in the evidence provided.

We are sincerely grateful for the constructive feedback provided by the reviewers. We believe that addressing these observations has substantially strengthened the manuscript's rigor and clarity. This study provides rare and essential empirical evidence on the psychological well-being of 'second-line' professionals in developing countries, bridging a critical gap for future mental health policy.
