# Supplementary material for: Social dysfunction in Ecuadorian, Peruvian, and Venezuelan media workers
Source: PLOS Ment Health. 2026 Sep 2;3(9):e0000709. doi: 10.1371/journal.pmen.0000709 (PMC13537591; doi:10.1371/journal.pmen.0000709)
Supplement: Peer review history

# Peer review: Decision Letter 0

**DOI:** 10.1371/journal.pmen.0000709.r001

**Authors:** Martin Mabunda Baluku

8 Feb 2026

PMEN-D-26-00016

Social dysfunction in ecuadorian, peruvian, and venezuelan media workers

PLOS Mental Health

Dear Dr. Granda,

Thank you for submitting your manuscript to PLOS Mental Health. After careful consideration, we feel that it has merit but does not fully meet PLOS Mental Health’s publication criteria as it currently stands. Therefore, we invite you to submit a revised version of the manuscript that addresses the points raised during the review process.

Please submit your revised manuscript by March 10, 2026. If you will need more time than this to complete your revisions, please reply to this message or contact the journal office at mentalhealth@plos.org. When you're ready to submit your revision, log on to https://www.editorialmanager.com/pmen/ and select the 'Submissions Needing Revision' folder to locate your manuscript file.

Please include the following items when submitting your revised manuscript:

- A letter that responds to each point raised by the editor and reviewer(s). You should upload this letter as a separate file labeled 'Response to Reviewers'.
- A marked-up copy of your manuscript that highlights changes made to the original version. You should upload this as a separate file labeled 'Revised Manuscript with Track Changes'.
- An unmarked version of your revised paper without tracked changes. You should upload this as a separate file labeled 'Manuscript'.

Guidelines for resubmitting your figure files are available below the reviewer comments at the end of this letter.

We look forward to receiving your revised manuscript.

Kind regards,

Martin Mabunda Baluku, Ph.D.

Academic Editor

PLOS Mental Health

Journal Requirements:

1. Please amend your detailed Financial Disclosure statement. This is published with the article. It must therefore be completed in full sentences and contain the exact wording you wish to be published.

i. Please clarify all sources of financial support for your study. List the grants, grant numbers, and organizations that funded your study, including funding received from your institution. Please note that suppliers of material support, including research materials, should be recognized in the Acknowledgements section rather than in the Financial Disclosure.

ii. State the initials, alongside each funding source, of each author to receive each grant. For example: "This work was supported by the National Institutes of Health (####### to AM; ###### to CJ) and the National Science Foundation (###### to AM)."

iii. State what role the funders took in the study. If the funders had no role in your study, please state: “The funders had no role in study design, data collection and analysis, decision to publish, or preparation of the manuscript.”

iv. If any authors received a salary from any of your funders, please state which authors and which funders.

If the reviewer comments include a recommendation to cite specific previously published works, please review and evaluate these publications to determine whether they are relevant and should be cited. There is no requirement to cite these works unless the editor has indicated otherwise.

Additional Editor Comments (if provided):

[Note: HTML markup is below. Please do not edit.]

Reviewers' comments:

Reviewer's Responses to Questions

**Comments to the Author**

1. Does this manuscript meet PLOS Mental Health’s publication criteria?

Reviewer #1: Yes

Reviewer #2: No

**********

2. Has the statistical analysis been performed appropriately and rigorously?

Reviewer #1: Yes

Reviewer #2: Yes

**********

3. Have the authors made all data underlying the findings in their manuscript fully available (please refer to the Data Availability Statement at the start of the manuscript PDF file)?

The PLOS Data policy

Reviewer #1: Yes

Reviewer #2: Yes

**********

4. Is the manuscript presented in an intelligible fashion and written in standard English?

Reviewer #1: Yes

Reviewer #2: No

**********

Reviewer #1: 1. Using a convenience sample may limit how well the findings represent the entire media population in these countries.

2. The study mentions "hyperconnectivity" as a risk factor but does not analyze how different digital platforms (like social media compared to traditional broadcasting) might affect this risk.

3. The manuscript assumes that social dysfunction does not have cases of "spontaneous recovery." However, it does not provide long-term data to support this claim for this specific group.

4. Some parts of the Results and Discussion should connect more clearly. For example, correlating the bivariate results with the final regression model could explain why certain demographic factors were excluded.

5. There should be a more thorough discussion of the limitations of convenience sampling and how it might have affected the reported 19.7% prevalence rate.

6. In the discussion about the need for objective, scalable tools for monitoring mental health. It is suggested to refer from the paper: “Mental Health Monitoring and Intervention Using Unsupervised Deep Learning on EEG Data”. This reference is important because it highlights the urgent need for objective, scalable, and accessible tools for early detection of mental health issues like depression and anxiety. While this manuscript uses self-report scales (GHQ-28), discussing advanced monitoring techniques such as EEG data analysis would strengthen the foundation by showing future directions for mental health monitoring beyond surveys. This would help connect traditional screening methods with emerging objective tools.

7. The assumption about the lack of spontaneous recovery needs to include references to existing long-term studies using the GHQ-28 Scale C or should state this as a limitation for future research.

8. The Results section should include a clearer explanation of the shift between Layer 1 and Layer 3 of the regression model. This will clarify why initial risk factors like "sex" and "age" were replaced by psychosocial factors.

9. The language in the Introduction should be refined to use more precise terms when discussing "trauma repair processes."

10. All references within the text must strictly follow the journal's citation style, especially regarding the brackets used in the reference list.

Reviewer #2: The manuscript needs editing to remove section that spontaneously to Spanish and oth secrions that are simply not clear. The paper does not account for the fact that younger, early-career journalists are more likely to take positions in rural or peripheral regions because these roles are less desirable, lower paid, higher risk, but offer entry into the profession and initial bylines. This factor may account fo the bulk of your findings and not be easy t chang.

**********

what does this mean?). If published, this will include your full peer review and any attached files.

**Do you want your identity to be public for this peer review?**  If you choose “no”, your identity will remain anonymous but your review may still be made public.

For information about this choice, including consent withdrawal, please see our Privacy Policy

Reviewer #1: No

Reviewer #2: No

**********

[NOTE: If reviewer comments were submitted as an attachment file, they will be attached to this email and accessible via the submission site. Please log into your account, locate the manuscript record, and check for the action link "View Attachments". If this link does not appear, there are no attachment files.]

# Author comment: Author response to Decision Letter 1

**DOI:** 10.1371/journal.pmen.0000709.r002

5 Mar 2026

Attachment Submitted filename: Response to Reviewers.docx

# Peer review: Decision Letter 1

**DOI:** 10.1371/journal.pmen.0000709.r003

**Authors:** Martin Mabunda Baluku

23 Mar 2026

PMEN-D-26-00016R1

Social dysfunction in Ecuadorian, Peruvian, and Venezuelan media workers

PLOS Mental Health

Dear Dr. Bustamante Granda,

Thank you for submitting your manuscript to PLOS Mental Health. After careful consideration, we feel that it has merit but does not fully meet PLOS Mental Health’s publication criteria as it currently stands. Therefore, we invite you to submit a revised version of the manuscript that addresses the points raised during the review process.

Ensure that the recommendations are not overstated and are based on the findings.

Please submit your revised manuscript by April 15, 2025. If you will need more time than this to complete your revisions, please reply to this message or contact the journal office at mentalhealth@plos.org. When you're ready to submit your revision, log on to https://www.editorialmanager.com/pmen/ and select the 'Submissions Needing Revision' folder to locate your manuscript file.

Please include the following items when submitting your revised manuscript:

- A letter that responds to each point raised by the editor and reviewer(s). You should upload this letter as a separate file labeled 'Response to Reviewers'.
- A marked-up copy of your manuscript that highlights changes made to the original version. You should upload this as a separate file labeled 'Revised Manuscript with Track Changes'.
- An unmarked version of your revised paper without tracked changes. You should upload this as a separate file labeled 'Manuscript'.

Guidelines for resubmitting your figure files are available below the reviewer comments at the end of this letter.

We look forward to receiving your revised manuscript.

Kind regards,

Martin Mabunda Baluku, Ph.D.

Academic Editor

PLOS Mental Health

**Journal Requirements:**

If the reviewer comments include a recommendation to cite specific previously published works, please review and evaluate these publications to determine whether they are relevant and should be cited. There is no requirement to cite these works unless the editor has indicated otherwise.

**Additional Editor Comments (if provided):**

Reviewer 2 makes important observations. I am convinced that the issues raised can be addressed.

These commends should be adequately addressed before the final decision can be made.

[Note: HTML markup is below. Please do not edit.]

Reviewers' comments:

Reviewer's Responses to Questions

**Comments to the Author**

Reviewer #1: All comments have been addressed

Reviewer #2: All comments have been addressed

**********

publication criteria?

Reviewer #1: Yes

Reviewer #2: No

**********

3. Has the statistical analysis been performed appropriately and rigorously?

Reviewer #1: Yes

Reviewer #2: Yes

**********

4. Have the authors made all data underlying the findings in their manuscript fully available (please refer to the Data Availability Statement at the start of the manuscript PDF file)?

The PLOS Data policy

Reviewer #1: Yes

Reviewer #2: Yes

**********

5. Is the manuscript presented in an intelligible fashion and written in standard English?

Reviewer #1: Yes

Reviewer #2: Yes

**********

**Reviewer #1:**  I recommend accepting the manuscript. The authors have thoroughly revised it, addressing all major reviewer comments and making necessary modifications. The revised version now meets the journal’s standards for quality, clarity, and ethics, and is ready for publication, offering a valuable contribution to the field.

1. The authors responded clearly and made major changes, refining the "hyperconnectivity" idea and fixing assumptions about spontaneous recovery.

2. The manuscript was carefully revised to improve language, remove non-English parts, and connect theory with results.

3. Layered binary logistic regression clarifies how distant social factors relate to psychological mechanisms.

4. This study offers vital evidence on social issues affecting underrepresented media workers in Latin America, filling a gap in global mental health research.

5. Adding interaction terms in Layer 4 increased model accuracy to 89.8%, giving clearer risk factor insights in urban areas.

**Reviewer #2:**  In this manuscript, the authors report on a survey of social dysfunction among media workers. Although the manuscript is more readable than earlier versions, with the prior problems of switching into Spanish and other unclear passages apparently corrected, the central interpretive weaknesses remain unresolved.

The main problem is that the data do not support the recommendations. The manuscript describes correlates of social dysfunction, but it does not establish the mechanisms underlying it, nor does it provide evidence linking the observed associations to the proposed interventions. As a result, the discussion moves beyond what the data can justify.

The authors report greater social dysfunction among younger participants, those with less training, poorer health, loneliness, self-blame, and concerns about their own health and about infecting family members. Presumably, this concern relates to COVID-19, but the timing of the survey in relation to the pandemic is not clearly stated and should be specified. Lower risk was observed among those living in capital cities and among those seeking emotional support or using humour as a coping strategy. However, these findings remain descriptive. They do not explain why social dysfunction occurs, nor do they identify a supported target for intervention.

A major interpretive flaw is the suggestion that limited access to social support explains the social dysfunction observed in 19.7% of the 314 individuals studied. The data do not demonstrate this. There is no evidence here that lack of social support is a causal factor. Variables such as loneliness, reduced help-seeking, and lower use of humour may simply reflect broader psychological capability or poorer overall mental health, mechanisms that are correlated with social dysfunction. The manuscript does not disentangle these possibilities.

Accordingly, the recommendations are not adequately grounded in the findings. There is no evidence presented that increasing the availability of social support would improve outcomes in this group. It is at least equally plausible that broader mental health difficulties are primary, in which case improving overall mental health would need to come first. The manuscript offers interventions, but provides no data indicating that these recommendations are likely to be effective.

In its current form, the paper overstates correlational findings and makes practical conclusions that are not supported by the evidence provided. Therefore, I cannot recommend its publication.

**********

what does this mean?). If published, this will include your full peer review and any attached files.

**Do you want your identity to be public for this peer review?**  If you choose “no”, your identity will remain anonymous but your review may still be made public.

For information about this choice, including consent withdrawal, please see our Privacy Policy

Reviewer #1: No

Reviewer #2: No

**********

[NOTE: If reviewer comments were submitted as an attachment file, they will be attached to this email and accessible via the submission site. Please log into your account, locate the manuscript record, and check for the action link "View Attachments". If this link does not appear, there are no attachment files.]

# Author comment: Author response to Decision Letter 2

**DOI:** 10.1371/journal.pmen.0000709.r004

12 Apr 2026

Attachment Submitted filename: Response_to_Reviewers_auresp_2.docx

# Peer review: Decision Letter 2

**DOI:** 10.1371/journal.pmen.0000709.r005

**Authors:** Annesha Sil

6 Aug 2026

PMEN-D-26-00016R2

Social dysfunction in Ecuadorian, Peruvian, and Venezuelan media workers

PLOS Mental Health

Dear Dr. Bustamante Granda,

Thank you for submitting your manuscript to PLOS Mental Health. After careful consideration, we feel that it has merit but does not fully meet PLOS Mental Health’s publication criteria as it currently stands. Therefore, we invite you to submit a revised version of the manuscript that addresses the points raised during the review process.

Reviewer 3 has requested some minor revisions - could you please revise your manuscript to address these?

Please submit your revised manuscript by Sep 05 2026 11:59PM. If you will need more time than this to complete your revisions, please reply to this message or contact the journal office at mentalhealth@plos.org. When you're ready to submit your revision, log on to https://www.editorialmanager.com/pmen/ and select the 'Submissions Needing Revision' folder to locate your manuscript file.

Please include the following items when submitting your revised manuscript:

- A letter that responds to each point raised by the editor and reviewer(s). You should upload this letter as a separate file labeled 'Response to Reviewers'.
- A marked-up copy of your manuscript that highlights changes made to the original version. You should upload this as a separate file labeled 'Revised Manuscript with Track Changes'.
- An unmarked version of your revised paper without tracked changes. You should upload this as a separate file labeled 'Manuscript'.

Guidelines for resubmitting your figure files are available below the reviewer comments at the end of this letter.

As the corresponding author, your ORCID iD is verified in the submission system and will appear in the published article. PLOS supports the use of ORCID, and we encourage all coauthors to register for an ORCID iD and use it as well. Please encourage your coauthors to verify their ORCID iD within the submission system before final acceptance, as unverified ORCID iDs will not appear in the published article. *Only*  the individual author can complete the verification step; PLOS staff *cannot*  verify ORCID iDs on behalf of authors.

We look forward to receiving your revised manuscript.

Kind regards,

Annesha Sil, Ph.D.

Staff Editor

PLOS

**Journal Requirements:**

If the reviewer comments include a recommendation to cite specific previously published works, please review and evaluate these publications to determine whether they are relevant and should be cited. There is no requirement to cite these works unless the editor has indicated otherwise.

Please review your reference list to ensure that it is complete and correct. If you have cited papers that have been retracted, please include the rationale for doing so in the manuscript text, or remove these references and replace them with relevant current references. Any changes to the reference list should be mentioned in the rebuttal letter that accompanies your revised manuscript. If you need to cite a retracted article, indicate the article’s retracted status in the References list and also include a citation and full reference for the retraction notice.

**Additional Editor Comments (if provided):**

[Note: HTML markup is below. Please do not edit.]

Reviewers' comments:

Reviewer's Responses to Questions

**Comments to the Author**

Reviewer #3: All comments have been addressed

**********

publication criteria?

Reviewer #3: Yes

**********

3. Has the statistical analysis been performed appropriately and rigorously?

Reviewer #3: Yes

**********

4. Have the authors made all data underlying the findings in their manuscript fully available (please refer to the Data Availability Statement at the start of the manuscript PDF file)?

The PLOS Data policy

Reviewer #3: Yes

**********

5. Is the manuscript presented in an intelligible fashion and written in standard English?

Reviewer #3: Yes

**********

**Reviewer #3:**  The authors have adequately addressed the comments raised during the previous round of review. The revised version shows important improvements in the clarity of the presentation, the organization of the manuscript, the description of the methodology, and the interpretation of the findings. Therefore, I consider that the manuscript meets the publication criteria of PLOS Mental Health and is now acceptable for publication.

The study addresses a relevant and still underexplored issue in the Latin American context by examining social dysfunction and its associated personal, occupational, and psychosocial factors among media workers in Ecuador, Peru, and Venezuela. The manuscript demonstrates consistency among the research problem, the objective, the methodological design, the instruments used, and the results obtained. It also provides sufficient information regarding ethical approval, informed consent, confidentiality, and participant anonymity.

The statistical analysis has been conducted appropriately and rigorously in relation to the study objective. The procedures are clearly explained, and the results adequately support the conclusions presented. The interpretation of the findings is consistent with the data and remains within the scope of a cross-sectional study. In addition, the discussion appropriately relates the results to previous literature and offers useful implications for the prevention of mental health difficulties and the development of psychosocial support initiatives for media workers.

According to the Data Availability Statement, the authors have made the information underlying the findings available within the manuscript and its Supporting Information files, including the supplementary dataset. Therefore, the manuscript complies with the PLOS data availability policy.

The manuscript is presented in a clear, coherent, and understandable manner. The English is generally sufficient to communicate the scientific content; however, a few minor issues related to grammar, wording, and terminological consistency remain and should be corrected through a final language review before publication. These issues do not affect the scientific soundness of the study or the overall understanding of the manuscript. Overall, I consider that the manuscript has improved substantially, satisfactorily addresses the previous comments, and represents a relevant contribution to the fields of occupational mental health and media workers’ working conditions in Latin America.

**********

what does this mean?). If published, this will include your full peer review and any attached files.

**Do you want your identity to be public for this peer review?**  If you choose “no”, your identity will remain anonymous but your review may still be made public.

For information about this choice, including consent withdrawal, please see our Privacy Policy

Reviewer #3: No

**********

[NOTE: If reviewer comments were submitted as an attachment file, they will be attached to this email and accessible via the submission site. Please log into your account, locate the manuscript record, and check for the action link "View Attachments". If this link does not appear, there are no attachment files.]

**Figure Resubmissions:**

# Author comment: Author response to Decision Letter 3

**DOI:** 10.1371/journal.pmen.0000709.r006

8 Aug 2026

Attachment Submitted filename: Response_to_Reviewers_auresp_3.docx

# Editor report: Decision Letter 3

**DOI:** 10.1371/journal.pmen.0000709.r007

**Authors:** Karli Montague-Cardoso

12 Aug 2026

Social dysfunction in Ecuadorian, Peruvian, and Venezuelan media workers

PMEN-D-26-00016R3

Dear Dr. Bustamante Granda,

We are pleased to inform you that your manuscript 'Social dysfunction in Ecuadorian, Peruvian, and Venezuelan media workers' has been provisionally accepted for publication in PLOS Mental Health.

Before your manuscript can be formally accepted you will need to complete some formatting changes, which you will receive in a follow up email. A member of our team will be in touch with a set of requests.

Please note that your manuscript will not be scheduled for publication until you have made the required changes, so a swift response is appreciated.

IMPORTANT: The editorial review process is now complete. PLOS will only permit corrections to spelling, formatting or significant scientific errors from this point onwards. Requests for major changes, or any which affect the scientific understanding of your work, will cause delays to the publication date of your manuscript.

If your institution or institutions have a press office, please notify them about your upcoming paper to help maximize its impact. If they'll be preparing press materials, please inform our press team as soon as possible -- no later than 48 hours after receiving the formal acceptance. Your manuscript will remain under strict press embargo until 2 pm Eastern Time on the date of publication. For more information, please contact mentalhealth@plos.org.

Thank you again for supporting Open Access publishing; we are looking forward to publishing your work in PLOS Mental Health.

Best regards,

Karli Montague-Cardoso

Staff Editor

PLOS Mental Health

***********************************************************

Reviewer Comments (if any, and for reference):
